# Cardiovascular Diseases: From Basic Research to Clinical Application

**DOI:** 10.3390/life15091399

**Published:** 2025-09-04

**Authors:** Cristiana Bustea, Delia Mirela Tit

**Affiliations:** 1 Department of Preclinical Disciplines, Faculty of Medicine and Pharmacy, University of Oradea, 410073 Oradea, Romania; cbustea@uoradea.ro; 2 Doctoral School of Biomedical Sciences, University of Oradea, 410087 Oradea, Romania; 3 Department of Pharmacy, Faculty of Medicine and Pharmacy, University of Oradea, 410028 Oradea, Romania

As we close the second edition of the *Life* Special Issue “Cardiovascular Diseases: From Basic Research to Clinical Application”, we would like to reflect on the progress made in understanding the molecular and physiological mechanisms underlying cardiovascular diseases and their implications in clinical interventions. The eleven papers featured in the first edition brought novel findings about heart failure, transcatheter aortic valve replacement, implantable cardioverter defibrillators, thrombosis, and coronary artery disease, which represent only a fraction of the vast field that is cardiovascular research. Now, the second edition presents a collection of 14 original studies, reviews, and case reports that highlight this continuum of discovery, from mechanistic insights to translational applications and clinical practice.

The summary below aims to captivate your attention by giving a glimpse into the Special Issue content. The manuscripts presented in this editorial are grouped according to the guest editors’ perspective on the papers, rather than strictly following their order of publication.

Cardiovascular diseases continue to be the leading cause of death [1], and research on better predictors, and means of prevention and treatment is of paramount importance. Despite major progress in the prevention and treatment of cardiovascular diseases, women remain an underdiagnosed and insufficiently treated group [2]. Research targeting this category of patients is expected to be performed in the future.

Heart failure represents a significant cause of increased rates of hospital readmissions, and its mortality and morbidity rates are still high [3]. Continuous research in the field seeks to develop new prognostic parameters to better tailor treatments to the specific conditions of each patient.

The original study by Catana A et al. [4], which opens the first edition of this Special Issue, provides proof that the mean platelet volume has a statistically significant relationship with negative predictor parameters such as NT-proBNP, reduced left ventricular ejection fraction, the presence of a dilated left ventricle, and pulmonary hypertension. Consequently, a new prognostic parameter could be added to existing ones, leading to an improved assessment of patients with heart failure and a more tailored treatment approach.

Interventional cardiology, since its debut in coronary revascularization, has changed the way we treat certain conditions that previously belonged to the surgical realm. One example is transcatheter aortic valve replacement, which has continuously developed, resulting in a younger patient population now eligible for the procedure [5]. Another example is implantable cardioverter defibrillators. The use of subcutaneous implantable cardioverter defibrillators in the prevention of sudden death by malignant arrhythmia represents a novel approach for young, active patients [6].

As the patients eligible for transcatheter aortic valve replacement (TAVR) are currently younger than before, coronary artery re-access following the procedure represents a main concern. In their review, Gherasie F.A. and Achim A. [7] outlined the strategies developed to protect against coronary obstruction during TAVR and ensure coronary artery re-access following the procedure. Moreover, the importance of commissural alignment and correct assessment of the risk factors for coronary artery occlusion during TAVR were emphasized, and the Basilica procedure and Chimney stenting procedure were thoroughly discussed.

Furthermore, in the comprehensive review by Guarracini F. et al. [8], the current state of subcutaneous implantable cardioverter defibrillators (sICDs) is extensively discussed. Although further research is needed, the improvements seen in the screening of patients before implant, the device implantation technique and programming, and the low risk of infection make sICDs a better option for well-selected patients.

Pathological coagulation, namely thrombosis, is a life-threatening condition. Although the coagulation cascade, once initiated, follows the same pathways, the origin of triggering factors is diverse. Conditions as different as COVID-19 [9], transcatheter interventions [10], and stent implantation [11] all share the same risk: the potential to trigger thrombosis. Uninterrupted research has been conducted on this topic, and progress has been seen in its management. However, there is still a long way to go and much room for improvement.

The COVID-19 pandemic has changed the world order. Although the pandemic has resolved, we still live with the disease and its consequences. Researchers have never ceased their work in this field as certain mechanisms are not yet fully understood. The original study by Baroni M. et al. [12] tackles the issue of coagulopathies induced by SARS-CoV-2 infection, bringing a new perspective to the management of this complication. In the authors’ opinion, identifying increased CD147 expression and utilizing papain-like protease inhibitors could serve as new tools in the approach of this condition.

The review by Singh R. et al. [13] belongs to the same area of thrombosis. It provides up-to-date information about the pathological activation of the coagulation cascade in thrombosis. The correlation between fibrinolytic activity in the body and the cardiovascular status of individuals was brought into discussion. The review emphasizes the importance of early recognition of increased fibrinolytic activity to prevent the development of cardiovascular diseases.

The success of transcatheter procedures is sometimes overshadowed by the occurrence of secondary thrombotic events. The manuscript by Preda A. et al. [14] reviewed the most recent studies about existing cerebral protection devices. Because none of the devices have shown a significant reduction in cerebral events, there is a need for randomized clinical trials and clear criteria to establish eligible patients.

The prevalent cause of morbidity and mortality is represented by coronary artery disease [15]. It can be acute or chronic, and despite the tremendous discoveries made in the field, more has to come. The introduction of coronary angiography has represented a big step forward in its treatment. Although stent implantation saves lives in patients with acute coronary syndromes, the risk of a recurrent event must not be overlooked and should be carefully assessed [16]. Chronic coronary artery disease, especially the non-obstructive form, has been underestimated in clinical studies due to its relatively low incidence of adverse outcome. However, multi-vessel non-obstructive coronary artery disease is associated with poor long-term prognostic [17], increasing the need for more intensive treatment. Research in this area is of current interest.

Coronary restenosis after balloon dilation is one of the possible complications. To prevent its occurrence, drug-coated balloons have been invented. In their original study, Katsouras C.S. et al. [18] demonstrated the non-inferiority of an innovative everolimus-coated balloon versus a sirolimus-coated balloon in an animal model. Although further research is needed to prove its biocompatibility, which apparently depends on the dose of everolimus, the current results are promising.

The paper by Nemes A et al. [19] is an original study regarding the role of three-dimensional echocardiography in the correct assessment of tricuspid annulus function. The study was performed on healthy adults without functional tricuspid regurgitation. By measuring the tricuspid annulus’s dimensions and functional sphincter-like properties, associated with TAPSE, the method demonstrated sensitive and harmonic tricuspid annulus function in the study group patients.

The second edition of the Special Issue opens with a wide-angle review [20] on the role of ectopic olfactory receptors in regulating the cardiovascular–kidney–metabolic axis.

The next study [21] presents a linear association between mucosal/gingival inflammation and carotid intima–media thickness values. Peri-implant mucosal inflammation appears to contribute to an individual’s vascular disease burden, but in the authors’ opinion, further studies are needed to validate this connection.

The original study by Busceti et al. [22] explored how methamphetamine induces neuroplastic changes, extending beyond the motor systems to alter autonomic regulation. Cardiovascular sensitization occurred independently of locomotor sensitization, and persistent blood pressure elevation may underlie specific mechanisms contributing to hypertension.

Though the presence of atherosclerotic coronary artery disease mitigates the effect of myocardial bridging on cardiovascular outcomes, the study by Yang et al. [23] suggests that myocardial bridging could be considered an insignificant equivalent of coronary artery disease.

Florindo et al. [24] investigated the restorative effects of regular physical activity in sedentary, multimorbid older adults. A simple 30-day home-based program effectively corrected lower limb perfusion asymmetries, reinforcing exercise as a cornerstone of vascular health promotion, even in vulnerable populations. Similarly, Mirjam Močnik and Nataša Marčun Varda [25] emphasized preventive measures to avoid further complications in hypertensive children.

Mitigating chemotherapy-induced atherosclerotic cardiovascular disease requires a comprehensive, multidisciplinary approach, as shown in the review by Izquierdo-Condoy et al. [26]. Establishing cardio-oncology units improves care coordination, and long-term surveillance allows for timely detection and intervention—both of which enhance cardiovascular outcomes and survivorship. The study by Militaru et al. [27] highlighted the need for accurate assessment of subclinical atherosclerosis and left ventricular function prior to chemotherapy. Complementing this, Kretsinin et al. [28] demonstrated the protective role of astaxanthin—a natural antioxidant—in cardiomyocytes exposed to oxidative stress and doxorubicin. By preserving mitochondrial mass, maintaining calcium homeostasis, and attenuating endoplasmic reticulum stress, their findings underscore the promise of nutraceuticals as adjunctive therapies in cardio-oncology.

Aortic valvular disease in its various clinical forms is addressed in three papers in this edition. Nemes et al. [29] studied the relationship between simultaneous end-diastolic and end-systolic left ventricular volumes and aortic valve area dimensions in healthy adults. Avakian et al. [30], focusing on sex-based differences, evaluated the prognostic value of left ventricular mass index in patients with symptomatic severe aortic stenosis. Salzillo et al.’s review [31] examined the association between bicuspid aortic valve and sudden death.

Two case-based contributions emphasize personalized care in complex cardiovascular disease. Cinezan et al. [32] described a patient with progressive carotid atherosclerosis despite optimal therapy, where adjunctive colchicine successfully stabilized the condition—highlighting the role of inflammation control beyond conventional lipid and antiplatelet regimens. Bustea et al. [33] reported on an elderly patient who developed complete atrioventricular block from the combined use of digoxin and beta-blockers, underscoring the dangers of polypharmacy in frail populations and the need for vigilance in managing drug interactions.

The second edition concludes with a study by Muste et al. [34] on the controversial topic of complete versus incomplete revascularization in patients with multivessel disease and non-ST-elevation acute coronary syndromes.

In conclusion, we hope that by offering insights into the manuscripts included in these two editions of the Special Issue, we have raised your interest in and desire to read these manuscripts. And, don’t forget that cardiovascular disease, with its diversity, represents a continually open field for research and improvement.
